# Distribution, habitat affinities and phenology of the *Micrargus herbigradus*-species group (Araneae: Linyphiidae) in Poland

**DOI:** 10.2478/s11756-018-0026-5

**Published:** 2018-03-19

**Authors:** Konrad Wiśniewski, Robert Rozwałka, Wanda Wesołowska

**Affiliations:** 1 Department of Zoology and Animal Physiology, Institute of Biology and Environmental Protection, Faculty of Mathematics and Natural Sciences, Pomeranian University in Słupsk, Arciszewskiego 22b, 76-200 Słupsk, Poland; 2 Department of Zoology, Faculty of Biology and Biotechnology, Maria Curie Skłodowska University, Akademicka 19, 20-033 Lublin, Poland; 3 Department of Biodiversity and Evolutionary Taxonomy, University of Wrocław, Przybyszewskiego 65, 51-148 Wrocław, Poland

**Keywords:** *Micrargus apertus*, *Micrargus georgescuae*, *Micrargus herbigradus*, Central Europe, New records, Mountains

## Abstract

We review the known information on the distribution and habitat affinities of the *Micrargus herbigradus*-species group in Poland. The analysis is based on a thorough literature survey, our own materials, and verification of some older collections. We give new diagnostic drawings and review the characters that are useful in identification of species within the group. Three species are present in Poland: *M. herbigradus* (Blackwall, 1854), *M. apertus* (O.-P. Cambridge, 1870) and *M. georgescuae* Millidge, 1976. The latter is recorded for the first time in the country, and we add numerous new localities for the two former species. *Micrargus herbigradus* is common and widespread in Poland, living in various habitats, with only a slight preference to forests. In contrast, *M. apertus* is widely distributed but rarely found, while its affinity to forests is the highest within the group. The records of this species are most numerous in lowland forests (up to c. 300 m a.s.l), but it can also be found at higher altitudes. *M. georgescuae* is found only in montane habitats, both in the Sudetes and the Carpathian Mountains, from above 650 m a.s.l. The adults of all three species occur the whole year round, but seem to be most abundant in May and June.

**Electronic supplementary material:**

The online version of this article (10.2478/s11756-018-0026-5) contains supplementary material, which is available to authorized users.

## Introduction

The *Micrargus herbigradus*-group contains four Central European species: *M. alpinus* Rëlys and Weiss, 1997, *M. apertus* (O.-P. Cambridge, 1870), *M. georgescuae* Millidge, 1976 and *M. herbigradus* (Blackwall, 1854). Two species from this group, *M. apertus* and *M. herbigradus*, have been recorded in Poland. Here we add one more species to the list of the Polish fauna, i.e. *M. georgescuae*. *Micrargus subaequalis* (Westring, 1851), which is also present in Poland, belongs to a separate species group.

The species group discussed here was revised by Millidge (1976), and subsequently one further species was described from the Alps by Rëlys and Weiss (1997). Due to difficulties in the identification of *Micragus* species, their actual distribution in Poland was uncertain. Some authors suggested the presence of more than two *herbigradus*-group members (Staręga and Kupryjanowicz 1996), or the presence of some new species (Staręga 2003a), but they did not make final conclusions about the specimens’ identity. Since the species are difficult to identify we summarize all the features that are useful for their determination.

Although the biology of this species group is fairly well described in the literature, there is no thorough analysis of the data from Poland. Some of the published remarks on their ecology might have been simplifications. Thus, we summarize the features for accurate species identification, revise the data on the distribution of these species in the country, referring both to published information and our own findings, and analyze their characteristic habitats and phenology. Furthermore, we review the ambiguous data from previous publications.

## Methods

We gathered information from different studies, both published and unpublished. The published data are listed in the results, for which some of the previous records were verified (Staręga and Kupryjanowicz 1996; Staręga 2003a). In total, 97 articles were used for summarizing information on the distribution, habitat preferences and phenology of the *herbigradus*-group in Poland. Additionally, we included unpublished records that emanate from our own research in the analysis, of which all the specimens were identified by us. The detailed list of new, unpublished records is attached as supplementary electronic material (Online Resource 1: Tables S1–S4). The analysis of the distribution is based on research utilizing diverse methods of sampling material, i.e. pitfall traps, sweep net, sieving or direct search. The intensity of research in different parts of the country was decisively uneven, thus some results might be biased.

For the analysis of the altitudinal distribution and habitat preferences, we used the single sampling plots as units and did not consider the numbers of recorded specimens. When different habitats were investigated in a particular study – even if they were situated very close to each other – we counted them as separate plots. Based on literature, we assigned each plot to an altitudinal interval at 100 m resolution, i.e. 0–100, 100–200 m a.s.l., etc. This resolution still allowed us to draw some meaningful conclusions, but it was the highest resolution that we could apply to data from the literature.

Furthermore, the properties of the habitats in each plot were studied. In the first step of the analysis we divided the habitats into open or forested. The second division was based on the humidity. We sorted habitats according to their humidity, with five successive levels. The attribution of each habitat to a humidity level does not refer to the exact place or time (which could not be verified), but to the typical features of a habitat type, e.g. all lowland alder forests or different mire types were recognized as ‘very moist’. The data in the literature were obviously of different quality or credibility, thus selected records were excluded from some counts. The exact number of records used in each analysis is given in the results.

For the analysis of phenology as a unit we have used a single record of a species in one plot, per year (i.e. records from the same plot and date coming from two different years were counted separately). We have divided the records into those coming from the first half of each month (1st–15th) and those from the second half (16th–end). For data from pitfall traps we have assigned the record to the respective period by taking the middle date of the trap exposure time.

## Results

### Distinguishing species

Males of the *Micrargus herbigradus*-group can be identified by comparing their embolus, size and form of the adjacent *lamellae*, and the shape of the process (apical spine) that accompanies the embolus. *Micrargus georgescuae* has a conspicuously wide basal part of the embolus (Figs. 4, 11), while that of *M. apertus* and *M. herbigradus* is thin (Figs. 17 and 19, respectively). The apical spine of the palp is slightly sinuous, with a truncated tip in *M*. *herbigradus* (Fig. 20), whereas *M. apertus* and *M. georgescuae* differ in having this process smooth apically (Figs. 18 and 12). This spine is shorter and straight in *M*. *apertus* (Fig. 18), but clearly longer and slightly bent in *M*. *georgescuae* (Fig. 12). The lamellae accompanying the process are much smaller in *M*. *georgescuae* (Fig. 15) compared to *M. apertus*, and have a distinctly different shape.

The females can be identified by the arrangement of the seminal ducts, a structure seen on the inner side of the epigyne after dissecting it. We suggest not to try distinguishing species without dissecting this structure, although there might be some interspecific differences (Figs. 21, 25, 28). The seminal ducts of *M. herbigradus* form very distinctive long, wide and bent coils in the anterior part of the epigyne, relatively far from the spermathecae (Figs. 29, 30), whereas in *M*. *georgescuae* the ducts form a small narrow loop (Figs. 23, 24). The ducts of *M. apertus* have a much more complex structure: the first loop extends clearly in the anterior part of the epigyne, and subsequently forms a posterior coil (Figs. 26, 27). We summarize the features useful in species identification in Table 1.

**Table 1:** Morphological characters useful in identification the *Micrargus herbigradus*-species group members

Character	*herbigradus* (Figs 19–20, 28–30)	*apertus* (Figs 16–18, 25–27)	*georgescuae* (Figs 1–15, 21–24)
Embolus basally	thin	thin	wide
Apical spine	short, sinusoid	short, straight	long, bent
Tip of apical spine	truncated	pointed	pointed
Lamella	robust, partially surrounding spine	large, rhomboid	small, triangular
Inlet part of seminal ducts, before the loop	short, straight	long, curved	short, straight
Anterior loop of seminal duct	loose, large, distant from spermatheca	narrow, small, close to spermatheca,	narrow, small, close to spermatheca
Distal part of seminal duct, behind the loop	long	short	short

**Figs 1–7:**
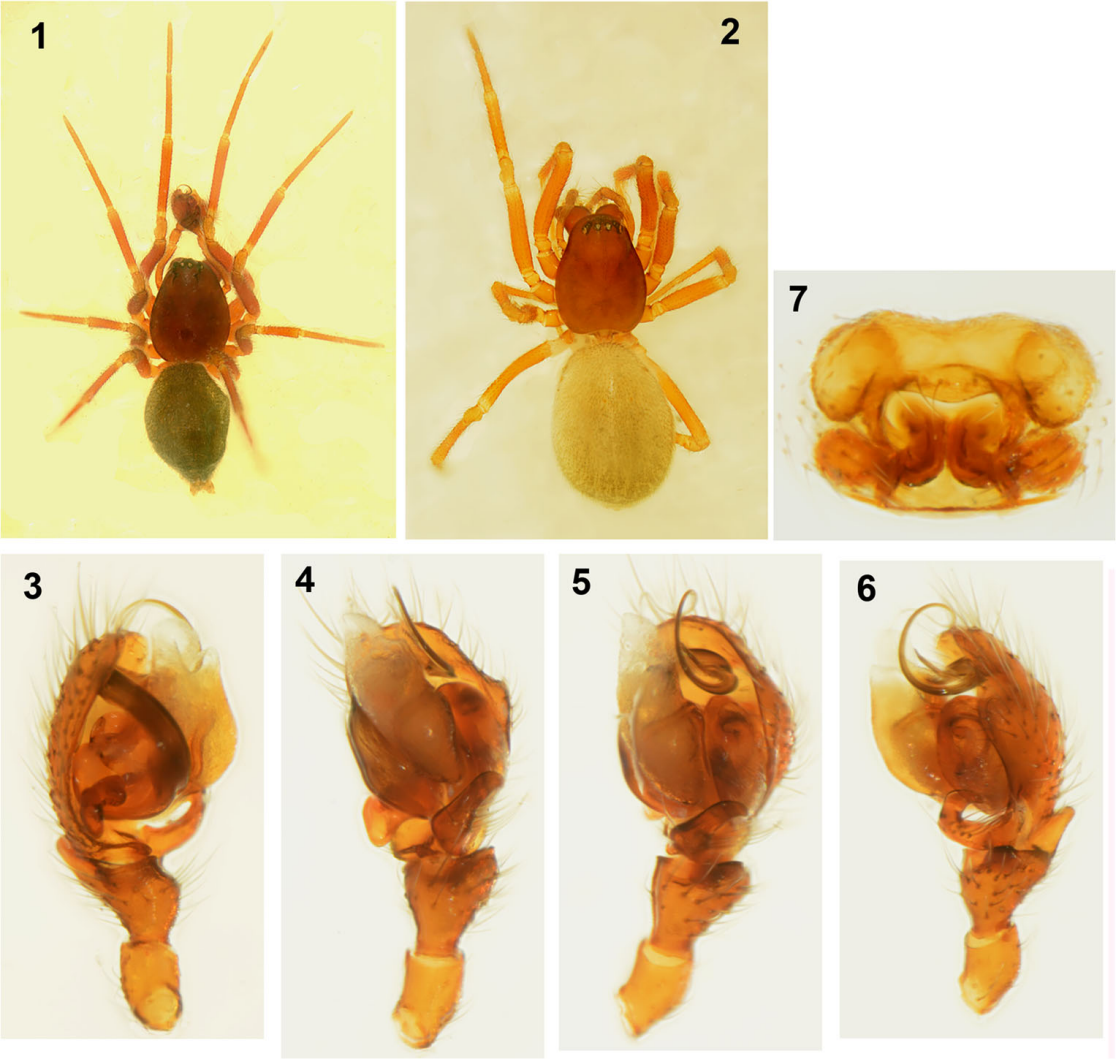
*Micrargus georgescuae* Millidge, 1976. 1. General appearance of male; 2. General appearance of female; 3. Palpal organ, prolateral view; 4. Ditto, ventral view; 5. Ditto, ventroretrolateral view; 6. Ditto, retrolateral view; 7. epigyne

The species differ also in the microsculpture of the stridulatory organs, which was shown by Rëlys and Weiss (1997). The other feature that distinguishes the *herbigradus*-group species is their size, *M. apertus* being the largest one, as mentioned by Millidge (1976). However, these characters are of little use in identifying spiders, unless one has all of the species available for comparison.

### New localities

This study represents the first report of *M. georgescuae* in Poland. In total, 126 individuals (63 males and 63 females) were collected in several mountain ranges of the Carpathians – the Beskid Wyspowy, the Tatra Mountains, the Gorce (all in S-Central Poland); in the Orawa-Nowy Targ Basin, at high altitudes; and in the Sudetes – the Stołowe Mountains and the Giant Mountains (SW Poland; Fig. 31).

**Figs 8–15:**
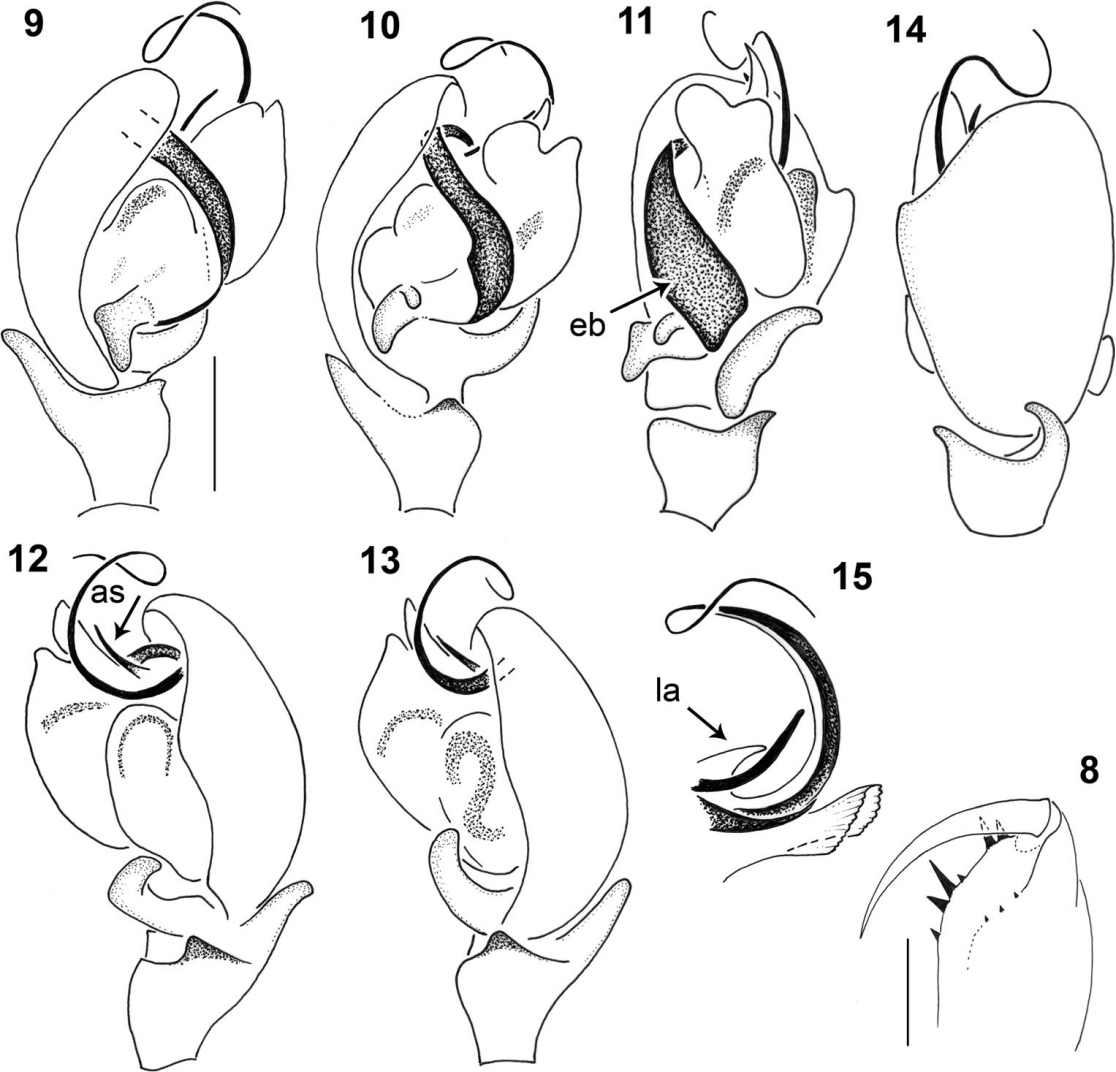
*Micrargus georgescuae* Millidge, 1976, male. 8. Cheliceral dentition; 9. Palpal organ, prolateral view; 10. Ditto, ventroprolateral view; 11. Ditto, ventral view; 12. Ditto, ventroretrolateral view; 13. Ditto, retrolateral view; 14. Ditto, dorsal view; 15. embolic part. Scales: 0.1 mm (9–14), 0.2 mm (8), eb - embolic base, as – apical spine, la – lamella

The specimens from the Gorce come from the study by Staręga and Kupryjanowicz (1996); most of this material belonged to *M. georgescuae* (Online Resource 1: Table S2). The species was present solely in the mountains, thus its range is restricted to the southern part of the country. It is not widespread, but in places where it lives, *M. georgescuae* may be quite numerous (Online Resource 1: Table S3). When one considers its broader European range, it was found in several major mountain ranges of Central Europe (see the Discussion).

*Micrargus apertus* is reported from five new localities in different parts of the country; it has already been recorded from 16 UTM (Universal Transverse Mercator) squares previously. The localities are scattered around the country, from the lowlands to the mountains (Fig. 32, Online Resource 1: Table S1).

**Figs. 16–20:**
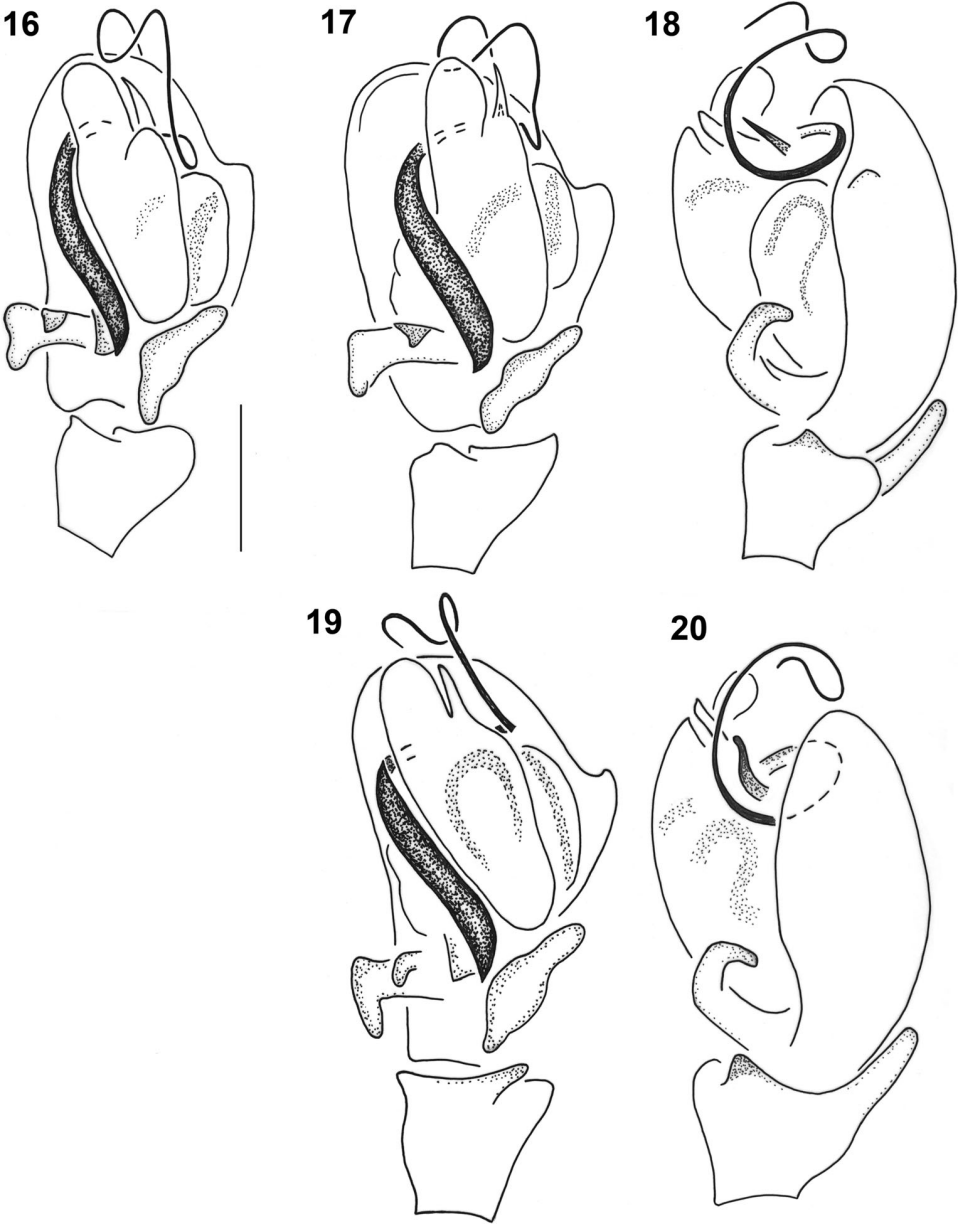
*Micrargus apertus* (O.-P. Cambridge, 1870) and *Micrargus herbigradus* (Blackwall, 1854), males. 16. *M. apertus*, palpal organ, ventral view; 17. Ditto, ventroretrolateral view; 18. Ditto, retrolateral view; 19. *M. herbigradus*, palpal organ, ventral view; 20. Ditto, retrolateral view. Scale: 0.1 mm

*Micrargus herbigradus* is extremely common, and the relatively large number of published and newly added localities from Poland does not totally depict its true distribution, which is surely much broader. In addition to the 175 UTM squares that it has been reported from previously, we add 40 points to the grid. We confirm its presence in eight localities. In total, the species has now been recorded from 215 UTM grid squares (Fig. 33; Online Resource 1: Table S4).

**Figs. 21–24:**
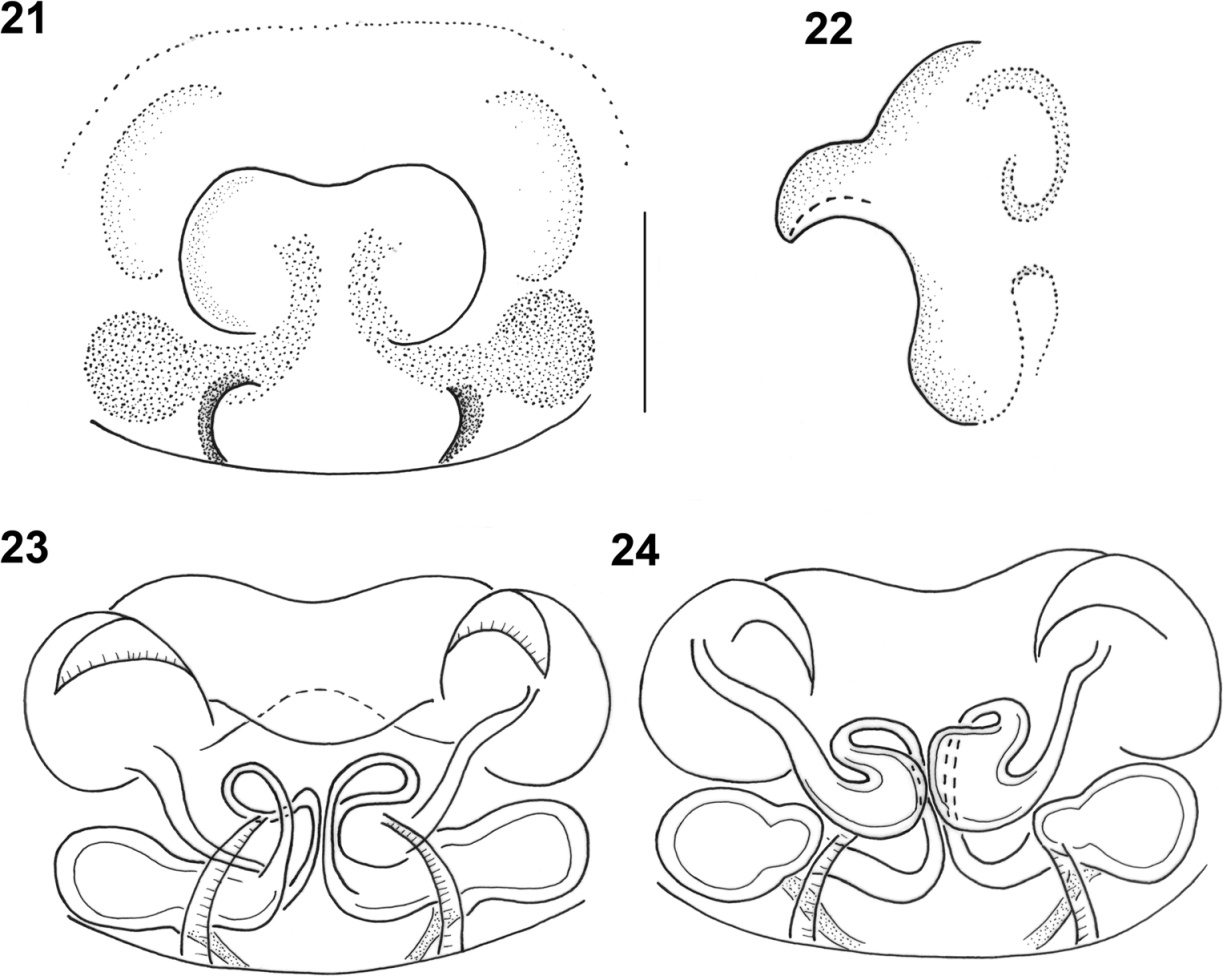
*Micrargus georgescuae* Millidge, 1976, female. 21. Epigyne, ventral view; 22. Epigyne, lateral view; 23. Spermathecae, ventral view; 24. Spermathecae, dorsal view. Scale: 0.1 mm

**Figs 25–30:**
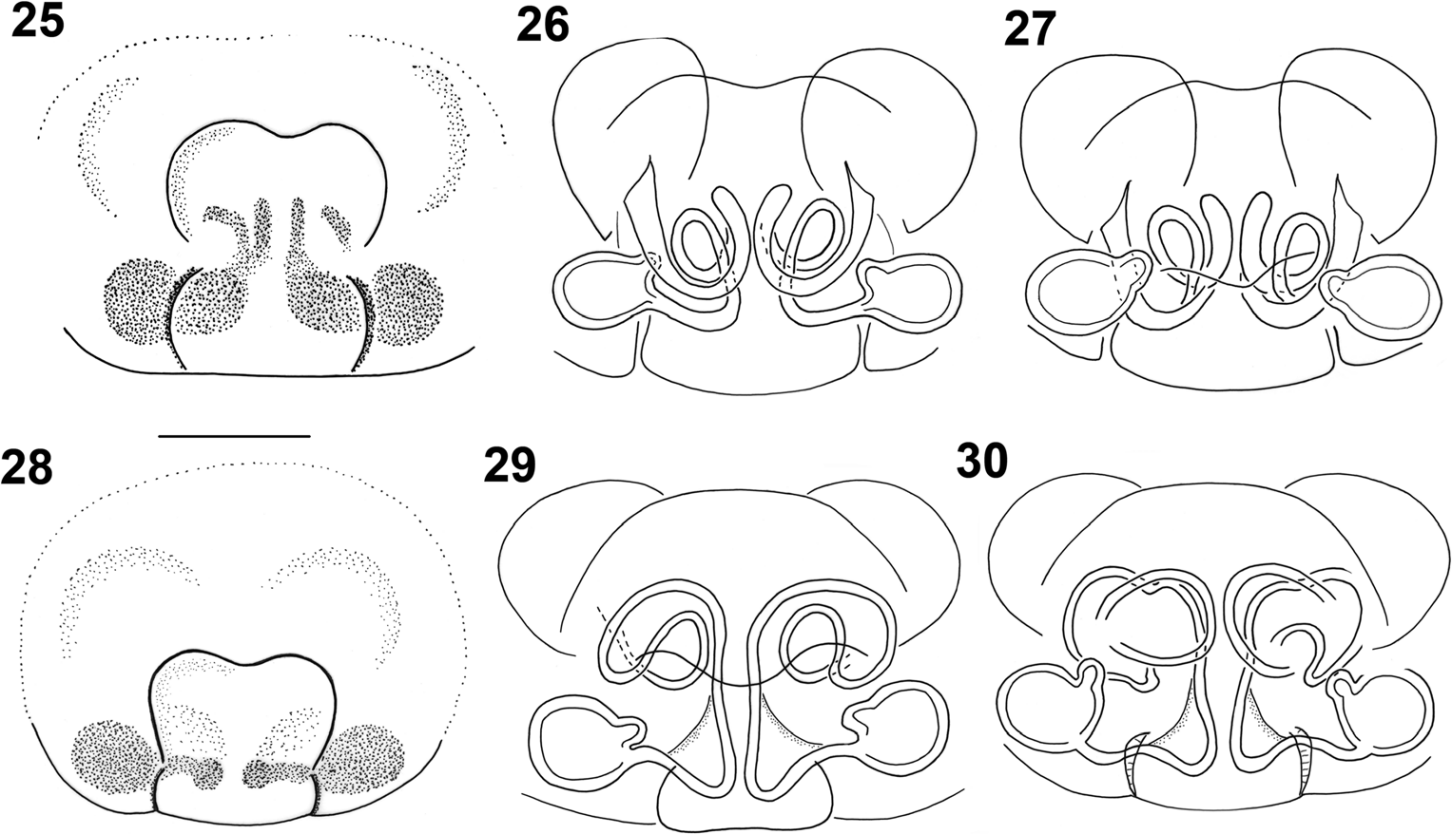
*Micrargus apertus* (O.-P. Cambridge, 1870) and *Micrargus herbigradus* (Blackwall, 1854), females. 25. *M. apertus*, epigyne; 26. Ditto, spermathecae, ventral view; 27. Ditto, spermathecae, dorsal view; 28. *Micrargus herbigradus*, epigyne; 29. Ditto, spermathecae, ventral view; 30. Ditto, spermathecae, dorsal view. Scale: 0.1 mm

**Fig. 31:**
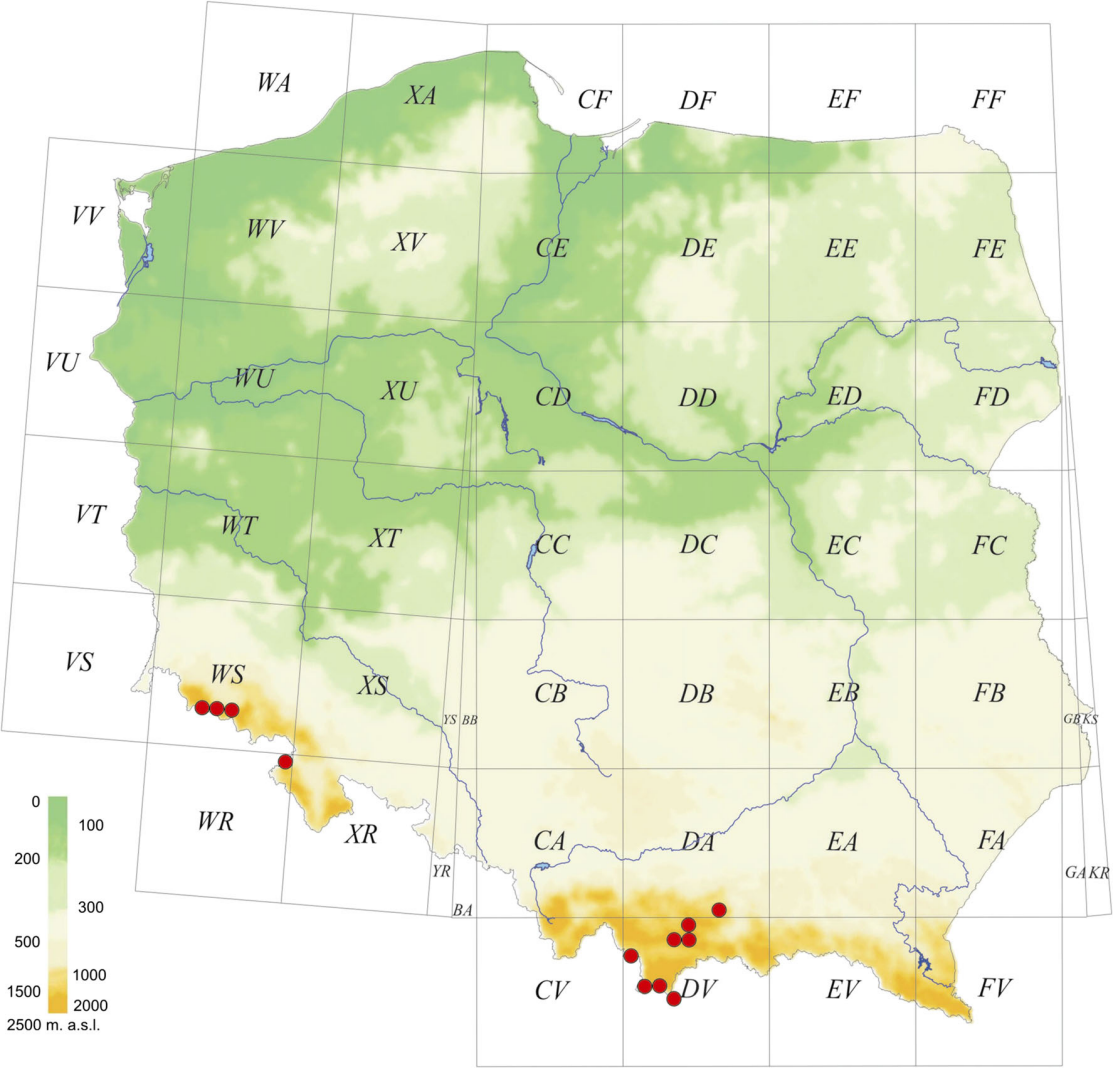
Distribution of *Micrargus georgescuae* in Poland. References: Staręga and Kupryjanowicz (1996): as a new species related to *Micrargus georgescuae* (only part of specimens). The red dots in all maps refer to our own findings or the material verified by us

**Fig. 32:**
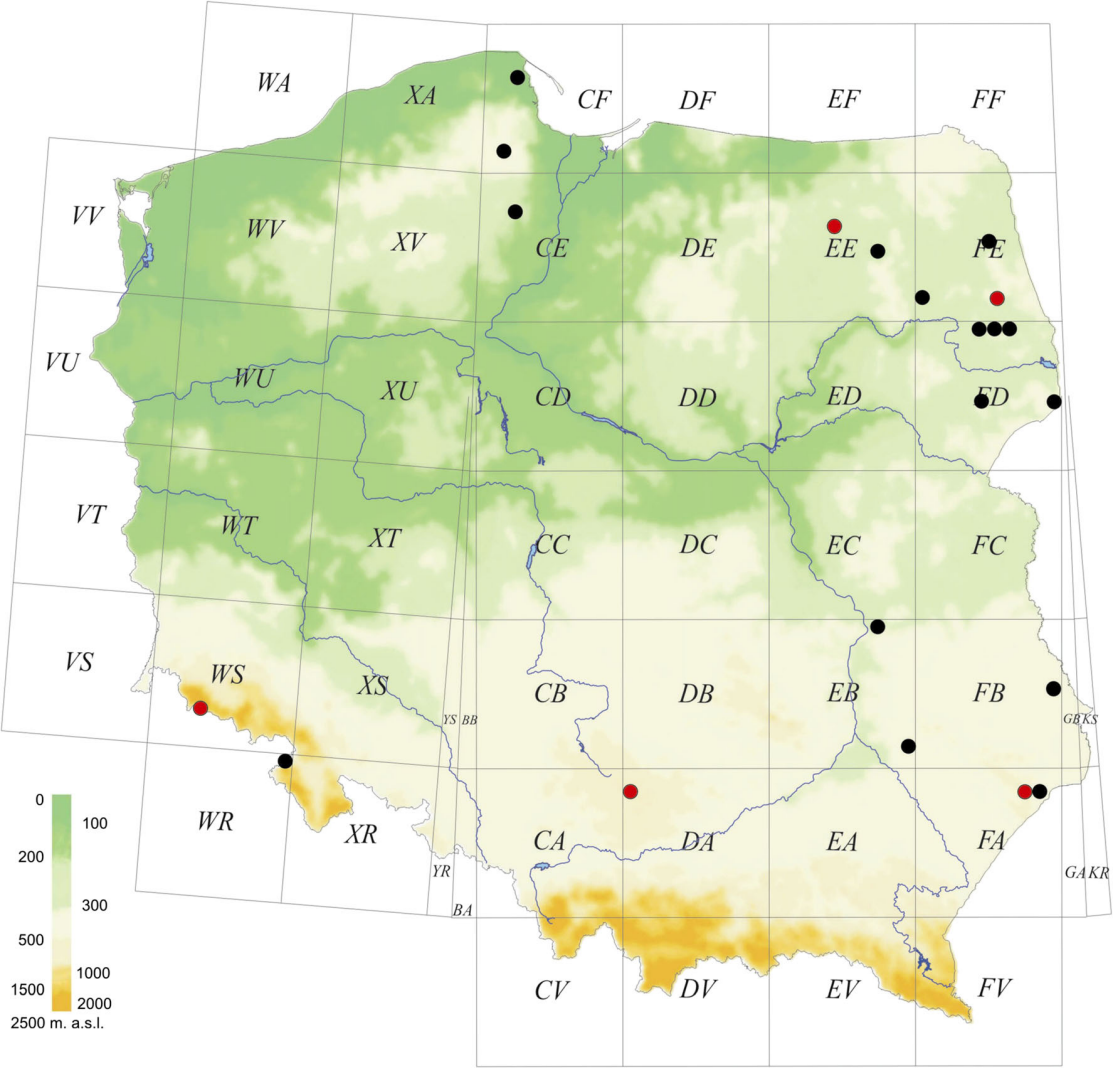
Distribution of *Micrargus apertus* in Poland. References: Staręga (1996a), Staręga and Stankiewicz (1996), Chyży and Staręga (1997), Staręga (2003a), Rozwałka (2004), Kupryjanowicz (2005), Stańska (2005, 2007), Rozwałka (2009, 2010a), Hajdamowicz et al. (2016). The red dots refer to our own findings, the black dots to the data from the literature

**Fig. 33:**
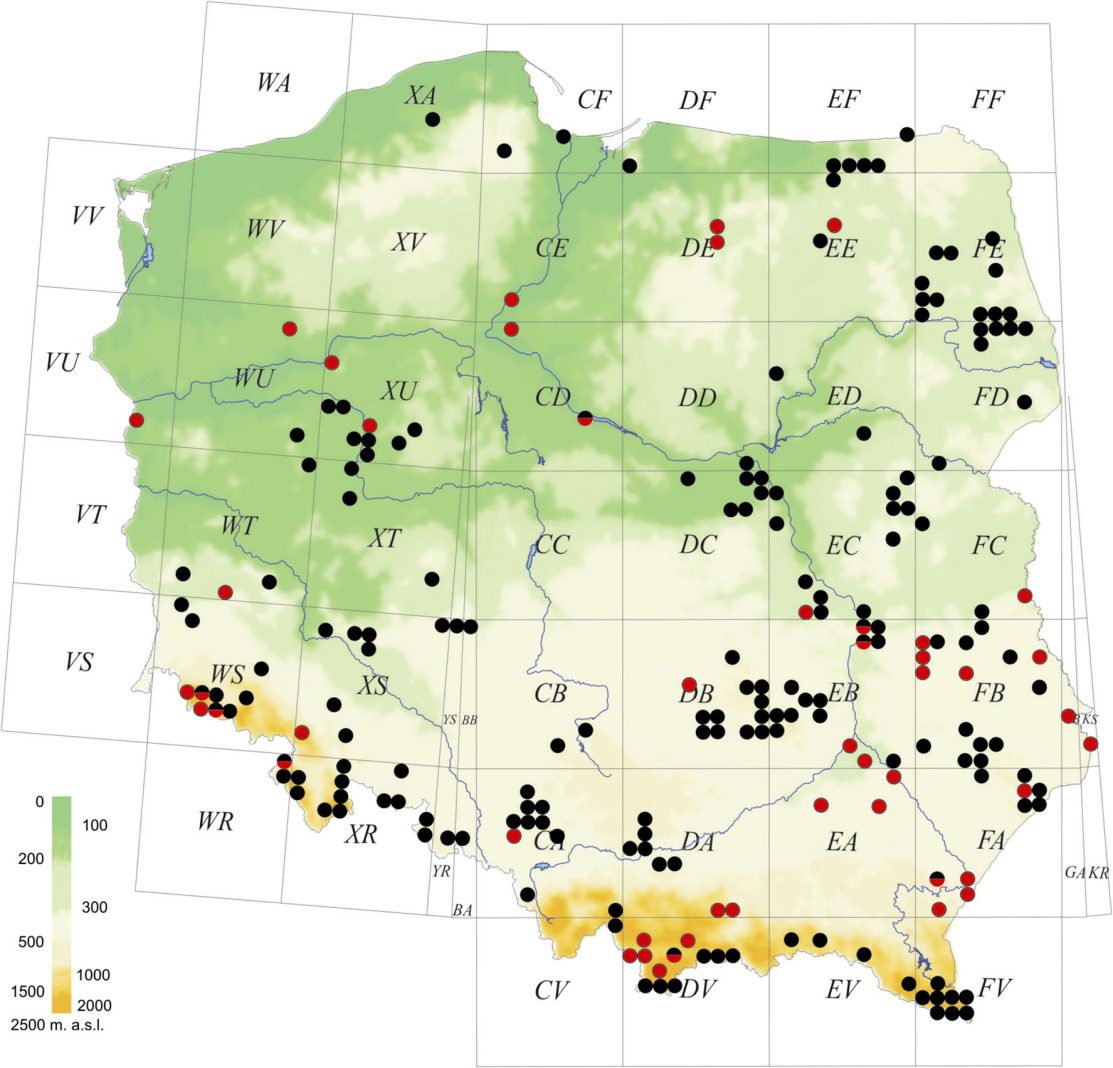
Distribution of *Micrargus herbigradus* in Poland. References: Nowicki (1874), Kulczyński (1876, 1881), Dahl (1902), Schenkel (1929), Kajak (1960), Łuczak (1960), Pilawski (1962), Sanocka-Wołoszyn (1964), Pilawski (1965), Bednarz and Czajka (1966), Pilawski (1966a, b), Staręga (1966), Pilawski (1967), Pilawski (1970), Prószyński and Staręga (1971), Staręga (1971, 1972), Deltshev and Kajak (1974), Dziabaszewski (1974), Staręga (1974), Woźny (1975a, b), Czajka (1976), Czajka and Goos (1976), Staręga (1976), Woźny (1976), Staręga (1978), Jędryczkowski and Staręga (1980), Krzyżanowska et al. (1981), Puszkar (1981), Sanocka-Wołoszynowa (1981), Czajka and Kornalewicz (1982), Krzyżanowska (1982), Staręga (1984), Woźny (1985), Staręga (1988), Tomek (1988), Woźny et al. (1988), Dziabaszewski (1989), Dziabaszewski et al. (1989), Staręga (1989), Dziabaszewski (1991), Woźny (1992), Czajka and Domin (1993), Wojtaczka and Woźny (1993), Łęgowski (1995), Staręga (1995), Sielicki and Staręga (1996), Staręga (1996a, b), Staręga and Stankiewicz (1996), Woźny (1996), Baldy and Woźny (1998), Łuczak and Woźny (1999), Szymkowiak et al. (1999), Rozwałka (2000), Staręga (2000), Woźny and Szymkowiak (2000), Łęgowski (2001), Staręga and Kupryjanowicz (2001), Baldy (2002), Wolak (2002), Stańska et al. (2002), Kupryjanowicz (2003), Staręga (2003a, b), Kajak and Oleszczuk (2004), Rozwałka (2004), Szymkowiak and Górski (2004), Wolak (2004), Kupryjanowicz (2005), Łęgowski (2006), Rozwałka (2006a, b, c), Rozwałka (2007a, b), Rozwałka 2008, Stańska and Łydkowska (2008), Rozwałka and Juszczyński (2009), Rozwałka (2009), Oleszczuk (2010), Rozwałka (2010a, b, c), Rozwałka (2012), Cichocki and Rozwałka (2013), Rozwałka (2014a, b), Rozwałka et al. (2014). Colours of the dots as in the Fig. 32. The red-black dots show the regions, where presence of the species was confirmed

**Figs 34–36:**
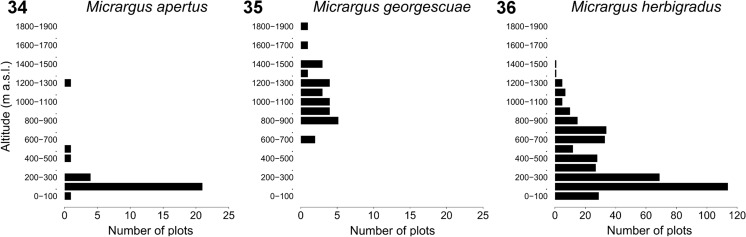
Altitudinal distribution of the species from the *Micrargus herbigradus*-group in Poland. Total number of plots: *M. herbigradus* – 390, *M. apertus* – 29, *M. georgescuae* – 28

**Figs 37–42:**
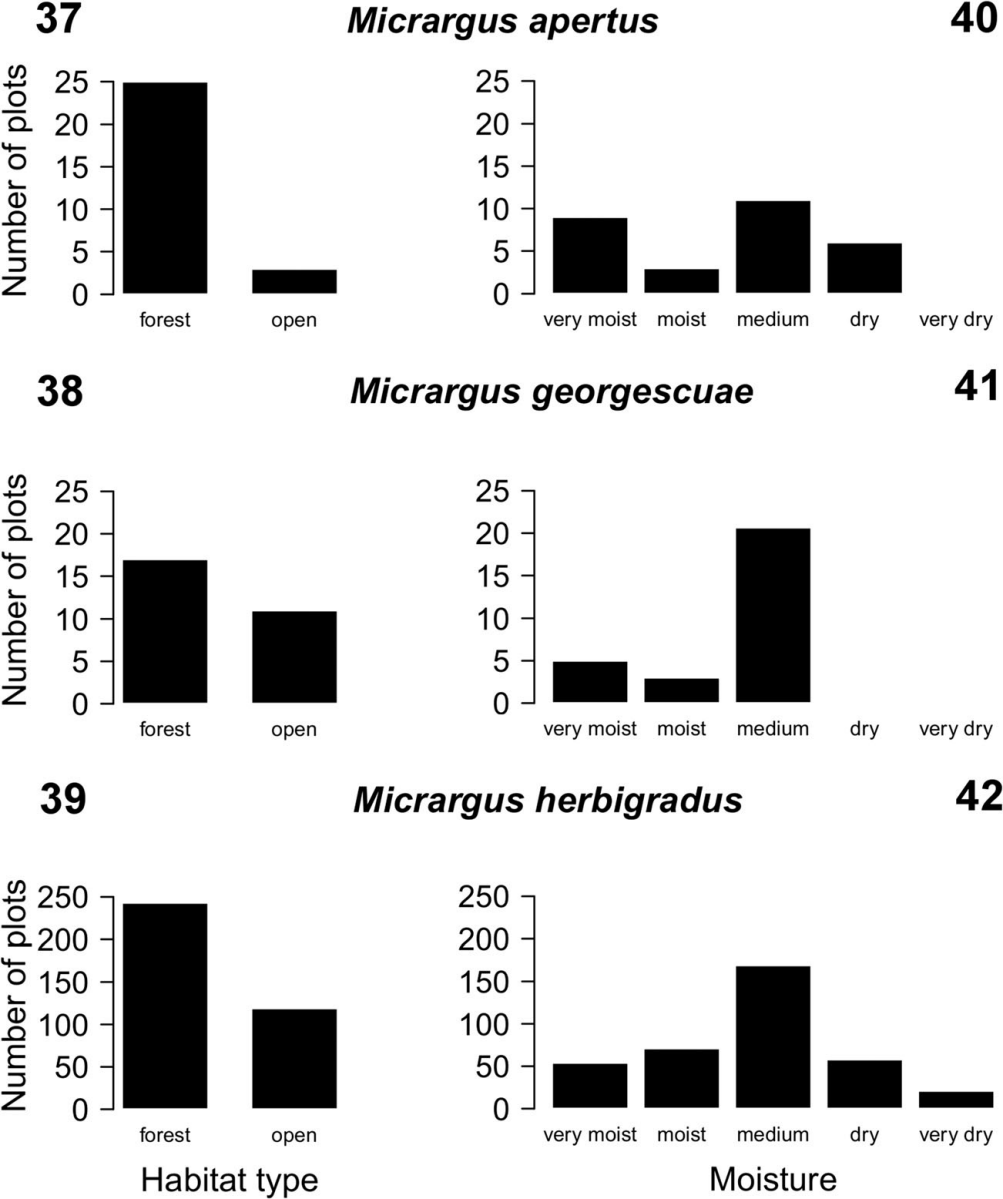
Number of forested or open plots with representatives of the *Micrargus herbigradus*-group in Poland and affinity of these species towards habitats of different moisture. Number of plots: *M. herbigradus* – 362, *M. apertus* – 28, *M. georgescuae* – 28 (habitat type); *M. herbigradus* – 373, *M. apertus* – 29, *M. georgescuae* – 28 (moisture)

**Figs 43–45:**
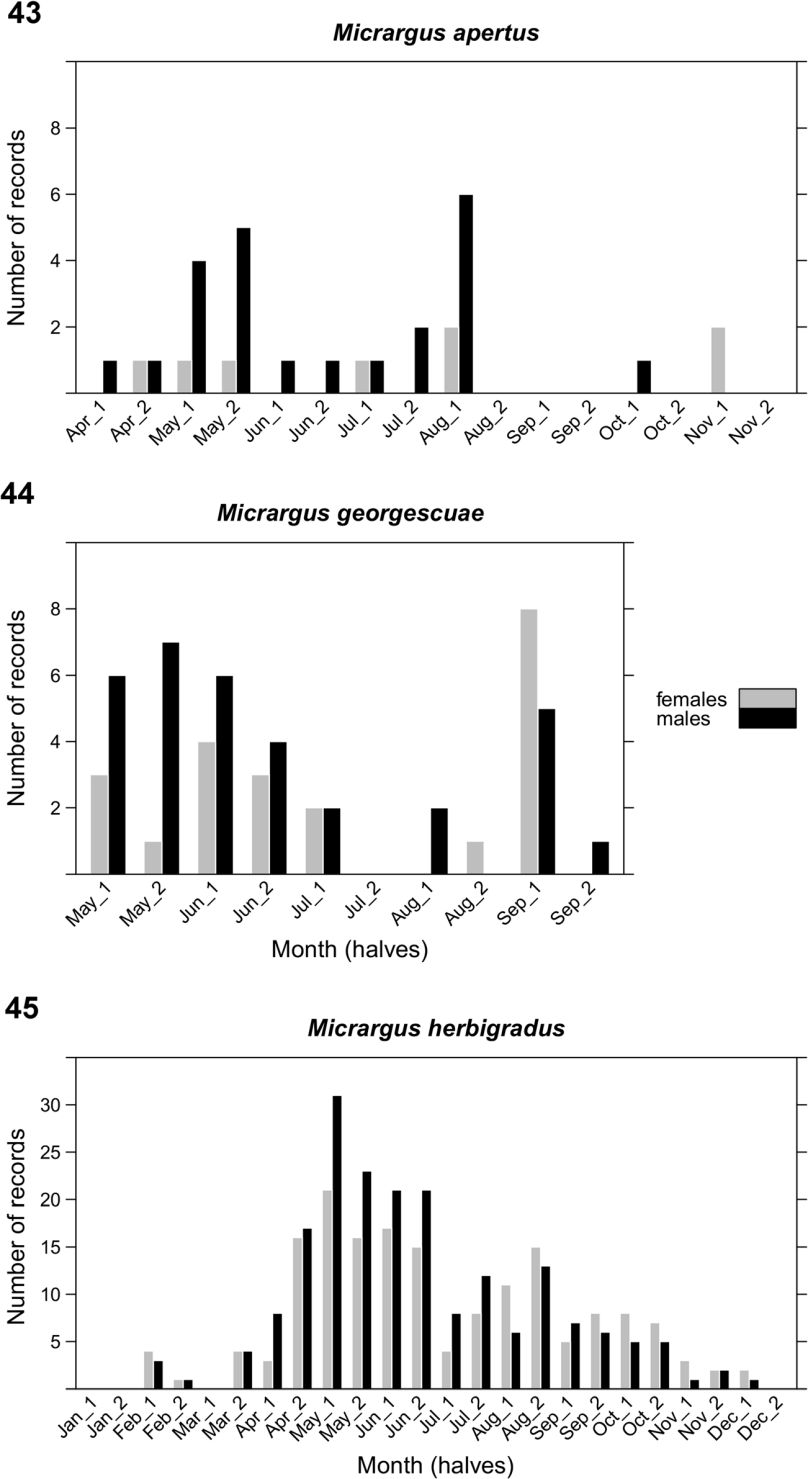
Temporal distribution of records within the *Micrargus herbigradus*-group in Poland. Number of records: *M. herbigradus* – 365, *M. apertus* – 31, *M. georgescuae* – 55 (males and females as separate records)

### Altitudinal distribution, habitats and phenology

The altitudinal distribution of the three species overlaps (Figs. 34–36). The only species with a clear preference to the mountains is *M. georgescuae* (Fig. 35), which in Poland occurs from about 670 m a.s.l., evenly distributed up to 1850 m a.s.l. (Online Resource 1: Tables S2, S3). *Micrargus apertus* and *M. herbigradus* were mostly found in Poland between 100 and 300 m a.s.l.. However, both of them appear in the mountains up to the subalpine level, and they sometimes cohabited with *M. georgescuae*.

All of the species show some preference towards forests compared to open habitats (Figs. 37–39), although they are not considered to be typical forest-dwellers. The percentage of the forested plots occupied by *M. apertus* was the highest of the three species, suggesting a stronger association with this habitat type (Fig. 37).

*Micrargus apertus* and *M. georgescuae* occur more frequently in humid habitats (Figs. 40, 41). The latter species is typical for the mountains, which – in Poland – have a cooler and more humid climate than in the other parts of the country. *Micrargus herbigradus* is present in all the habitat types (Fig. 42), from xerothermic, extremely dry stands, to periodically flooded forests and the mires. However, the majority of records come from stands of moderate moisture. The habitats of *M. apertus* are also diverse, including former sand quarries, crops, different forest types, caves and a subalpine bog. The diversity of *M. georgescuae*’s habitats is also conspicuous, i.e. montane spruce, alder or beech forests, dwarf pine shrubs, meadows, mountainous grasslands or different mire types.

The adults of the three species are present for most of year. *Micrargus apertus* was recorded from April to November. The data on its occurrence are scarce, but evenly distributed within the period (Fig. 43). The adults of *M. georgescuae* have two activity peaks, in late spring and early autumn (Fig. 44). The temporal distribution of *M. herbigradus* spans the whole year, with the highest peak in May and June (Fig. 45).

## Discussion

Our data update information on the distribution of the *M. herbigradus*-species group in Central Europe. *Micrargus georgescuae* is newly recorded from Poland, where it occurs in the mountains and is locally abundant. It inhabits several European mountain ranges, as the Alps (Thaler 1978; Maurer and Walter 1980; Rëlys and Weiss 1997; Höfer et al. 2010), but also various lower massifs in Germany (Arachnologische Gesellschaft 2017), Czechia (Buchar and Růžička 2002; The Czech Society of Arachnology 2017) and Slovakia (Franc 2002; Svatoň and Kovalčík 2006). It is also present in some other mountain ranges of the Carpathians (Gajdoš et al. 2014), namely in the Ukrainian Chornohora (Hirna et al. 2016) and in Romania (Georgescu 1971; Millidge 1976; Urák and Samu 2008). The species has already been recorded in the mountains close to the study sites referred to in the text, such as the Ore Mountains (Růžička and Hajer 2000), the Hrubý Jeseník (Majkus 2006) or the Great Fatra (Franc 2002), but also in the same mountain ranges, i.e. the Giant Mountains (Kůrka and Vaněk 2001; Kůrka and Vaněk 2009; Materna et al. 2010), the Central Sudetes (Buchar and Růžička 2002) and the Tatra Mountains (Svatoň and Kovalčík 2006). *Micrargus georgescuae* was not found in the mountains of SE Poland (the Bieszczady and the Eastern Beskidy), even though these ranges have been very intensively surveyed recently. It is also absent in the Slovak part of this mountain massif (Svatoň et al. 2003 only list *M. herbigradus*). Similarly, *M. georgescuae* was not found in the Izera Mountains (the Western Sudetes) that are close to the other mountain ranges where the species occurs. These mountains have also been intensively surveyed during the last decade.

*Micrargus apertus* is a species that is rarely found. Considering the arrangement of its localities in other countries, e.g. in Germany (Arachnologische Gesellschaft 2017) or the Czech Republic (Czech Society of Arachnology 2017), and the uneven, apparently disordered distribution in Poland, it might be expected that *M. apertus* is much more widespread, although uncommon. On the British Islands it seems to be more common in the northern parts (British Arachnological Society 2017). The species might often have been misidentified. *Micrargus herbigradus* is widespread and common almost throughout Europe (Nentwig et al. 2017), and the presented distribution map of Poland is surely incomplete.

The results on the altitudinal preferences of the three species in Poland might be biased both by the geography of the country, with its area situated mostly at the altitudes between 0 and 300 m a.s.l., and by the way the arachnologists have chosen investigated regions. There are numerous gaps, i.e. regions that have received little or no interest historically (e.g. the Western Pomerania and some parts of Central Poland). There is a clear affinity of *M. georgescuae* towards mountainous regions, with the lowest localities situated approximately at 650 m a.s.l. A similar pattern might be observed in Germany, where the species clearly prefers mountains (Arachnologische Gesellschaft 2017). However, in the Czech Republic the spider was found at lower altitudes, from 350 m a.s.l., with a peak around 400–600 m (Buchar and Růžička 2002). It might be linked to abundance of montane areas in this country and the availability of specific habitats such as wetlands or stone gorges, with which the species might by partly associated there (Buchar and Růžička 2002). Altitudinal ranges of the species from the *herbigradus*-group overlap both in Poland, in the Czech Republic (Buchar and Růžička 2002) and – for example – the Alps (Muster and Leipold 2001). In the latter case the authors mentioned some separation of the habitats within the group (additionally with *M. alpinus*), although sometimes the species were observed together (Muster and Leipold 2001). We have also observed their cohabitation in some localities. *Micrargus* is therefore an example of the genera that undergo high diversification in Europe, with a few examples of altitudinal vicariants, such as *M. alpinus* or *M. georgescuae*. Care should be taken in investigating material from the mountains, because some other new forms might be expected in the other massifs.

*Micrargus herbigradus* was sometimes described as a silvicolous species (Kupryjanowicz 2008; Nentwig et al. 2017), only sporadically occurring in open habitats (Buchar and Růžička 2002). However, this might be a broad simplification, or its preferences are geographically changeable, according to – for instance – the availability of specific habitat types in the region. In Poland and some other European countries (Hänggi et al. 1995) *M. herbigradus* is a common, eurytopic species, with no specific habitat preference. Generally, it is commoner in humid habitats and has no preference towards open or shaded places (Entling et al. 2007). In Poland, *M. apertus* seems to have an affinity towards shaded habitats. However, the total number of its records is considerably low, which may have influenced the results. *Micrargus georgescuae* does not have special habitat preferences, apart from living in cool and humid climates of the mountains.

*Micrargus herbigradus* also inhabits some specific microhabitats (Online Resource 1: Table S4), as we have observed this species in the (slightly destroyed) nest of the ant *Lasius fuliginosus* in a tree. It is also a common cave-dweller (Sanocka-Wołoszynowa 1964, Sanocka-Wołoszynowa 1981). The species was observed in the diet of dunnock hatchlings (*Prunella modularis*) by Tomek (1988).

The results on the phenology of the *M. herbigradus*-species group are consistent with those from some other studies. *Micrargus apertus* is recorded in almost all of the seasons (British Arachnology Society 2017), with the highest abundance from May to October (Arachnologische Gesellschaft 2017; Czech Society of Arachnology 2017), while *M. georgescuae* was recorded mostly from April to October (Czech Society of Arachnology 2017), similar to our study. One of our records of *M. georgescuae* comes from pitfall traps left for the duration of winter, from October to April (Online Resource 1: Tables S2, S3). Some species with winter activity of adults may be detected by leaving the traps beneath the snow cover. The adults of *M. herbigradus* can be found all year round, with a peak between May and July (Arachnologische Gesellschaft 2017; British Arachnological Society 2017; Czech Society of Arachnology 2017). In selected studies – some of which we had to exclude from the analysis – the authors observed two abundance peaks in late spring and in autumn (Staręga 2000, 2003a), or recorded considerable winter activity of this *Micrargus* species (Woźny 1992).

Our review on the *Micrargus herbigradus*-species group is one of the first works of its kind that deals with the spider fauna of Poland. It shows that there is still a need to clarify data on spider distribution and ecology, and to revisit published works. The knowledge of spiders is dispersed in various articles, predominantly only of local importance, and reviewing their information will enable more thorough analysis and allow them to be considered within a broader European context.

## Electronic supplementary material

ESM 1 **Table S1** New localities of *Micrargus apertus.* **Table S2** Verified localities of *Micrargus georgescuae.* **Table S3** New localities of *Micrargus georgescuae.* **Table S4** New localities of *Micrargus herbigradus.* (PDF 50 kb)
