# Supplementary material for: Distribution, habitat affinities and phenology of the Micrargus herbigradus-species group (Araneae: Linyphiidae) in Poland
Source: Biologia (Bratisl). 2018 Mar 19;73(2):151–64. doi: 10.2478/s11756-018-0026-5 (PMC5882768; doi:10.2478/s11756-018-0026-5)
Supplement: Supplementary file 1 — Table S1 New localities of Micrargus apertus. Table S2 Verified localities of Micrargus georgescuae. Table S3 New localities of Micrargus georgescuae. Table S4 New localities of Micrargus herbigradus. (PDF 50 kb) [file 11756_2018_26_MOESM1_ESM.pdf]

Online Resource 1

Title: Distribution, habitat affinities and phenology of the *Micrargus herbigradus*-species group (Araneae: Linyphiidae) in Poland

Journal: Biologia

Authors: Konrad Wiśniewski[1], Robert Rozwałka, Wanda Wesołowska

[1]Corresponding author: Department of Zoology and Animal Physiology, Institute of Biology and Environmental Protection, Faculty of Mathematics and Natural Sciences, Pomeranian University in Słupsk, 76-200 Słupsk, Arciszewskiego 22b, Poland; e-mail: konwisniew@gmail.com

Table S1. New localities of *Micrargus apertus* (ordered eastwards). UTM – Universal Transverse Mercator, m – males, f – females

Geographic area	Locality	GPS Coordinates	UTM Square	Altitude (m a.s.l.)	Habitat	Dates (exposure time for pitfall traps or precise date)	Total number m/f
Giant Mts	Kamiennik/Szrenica pass	50°47'21" N; 15°30'01" E	WS32	1250	subalpine bog	28.05–12.06.2010<br>07–27.05.2012	2/0
Kraków-Wieluń Upland	Psia Cave by Smoleń	50°25′35″ N; 19°39′58″ E	DA08	410	cave	17.05.2015	1/1
Masurian Lakeland	vicinity of Urwitałt	53°48'29" N; 21°39'13" E	EE46	140	mixed forest with dominance of pine	10.04–03.05.2007	0/1
Puszcza Knyszyńska (Knyszyn Forest)	Jesionowe Góry Reserve	53°20′ N; 23°17′ E	FE51	165	mixed forest with dominance of spruce	02.07.1999	0/1
Roztocze	by Bełżec	50°23′01″ N; 23°24′19″ E	FA78	278	former sand quarry, young pine trees	16.05–16.06.2005	1/0

Table S2. Verified localities of *Micrargus georgescuae* (ordered eastwards). UTM – Universal Transverse Mercator, m – males, f – females

Geographic area	Locality	GPS Coordinates	UTM Square	Altitude (m a.s.l.)	Habitat	Date	Total number m/f
Gorce Mts	Lepietnica Valley	49°32′ N; 20°04′ E	DV38	900	mixed forest with spruce and fir	7.05.1992	3/7
	Łopuszna Valley	49°32′ N; 20°07′ E		1200	beech forest	8.05.1992	1/0
	Jaworzyna Kamienicka	49°32′ N; 20°09′ E	DV39	1280	upper zone spruce forest	4.09.1986	0/2
		49°33' N; 20°09′ E		960		7.05.1992	2/0
				1100		2.06.1986	1/3
				1000		4.06.1986	0/7
	Jaszcze Valley	49°32' N; 20°11′ E	DV48	850	spruce forest	3.06.1986	0/1
				900		4.09.1986	1/1
				850	beech-sycamore forest	2.09.1986	3/6
	Polana Szlagowa	49°31' N; 20°11' E		1050	low spruce and meadows	3.09.1986	2/1
	Borsuczyny			1090	upper zone spruce forest	3.09.1986	5/17
				900	beech forest	3.09.1986	0/2
	Jaszcze Małe	49°32′ N; 20°12′ E		850	spruce forest	1.06.1986	1/0
				810	beech forest	5.09.1986	0/3

Table S3. New localities of *Micrargus georgescuae* (ordered eastwards). UTM – Universal Transverse Mercator, m – males, f – females

Geographic area	Locality	GPS Coordinates	UTM Square	Altitude (m a.s.l.)	Habitat	Dates (exposure time for pitfall traps or precise date)	Total number m/f
Giant Mts	Kocioł Szrenicki	50°47'17" N; 15°31'50" E	WS32	1170	sloping poor fen	26.05–16.06.2010	1/0
						16.06–06.07.2011	1/0
						10–30.05.2012	1/0
	by Przełęcz Karkonoska	50°47′ N; 15°36′ E	WS42	1110	spruce forest in upper mountain zone, 90-years old	8.06-15.07.2005	1/0
						9.06-15.07.2005	0/1
	by Pielgrzymy rocks	50°46'04" N; 15°41'15" E	WS42	1215	sloping poor fen	29.05–11.06.2010	1/0
						26.04–07.05.2011	1/1
						12.06–01.07.2011	1/0
						07–27.05.2012	1/0
	slopes of Smogornia	50°44'52" N; 15°41'18" E	WS42	1410	subalpine bog	26.05–15.06.2010	1/0
						04–26.07.2010	1/0
						28.04–11.05.2011	1/0
						11–31.05.2011	4/0
						17.06–06.07.2011	0/1
						11–31.05.2012	1/1
	Równia pod Śnieżką	50°44'23" N; 15°42'41" E	WS52	1430	Dwarf mountain pine and *Nardus stricta* grasslands	11–31.05.2012	7/0
						31.05–15.06.2012	0/2
						15.06–05.07.2012	0/2
						05–24.07.2012	0/1
						13.08–01.09.2012	0/1
	Kopa	50°44'45" N; 15°43'39" E	WS52	1370	subalpine bog	26.05–15.06.2010	3/0
						24.07–13.08.2010	1/0
						11–31.05.2011	2/0
						11–31.05.2012	6/0
Stołowe Mts	Szczeliniec Wielki	50°29' N; 16°20' E	WR99	895	moss in rocky crevice	1.08.2006	1/0
Orawa-Nowy Targ Basin	'Pustać' by Chyżne	49°24′45″ N; 19°43′41″ E	DV07	692	peat bog, hollow	20.10.2008-4.04.2009	1/0
Tatra Mts	Chochołowska Valley	49°15′ N; 19°50′ E	DV15	1010	subalpine meadows	18.06.1975	1/0
	vincity of Przełecz pod Kopą Kondracką	49°14′04″ N; 19°56′30″ E	DV25	1850	alpine meadow	5.09.2013	0/1
	Wielki Kopieniec	49°16′16″ N; 20°00′58″ E	DV25	1280	rocky, thermophilous montane grasslands	13.07.2014	1/1
	by Czarny Staw Pod Rysami	49°11′19″ N; 20°04′49″ E	DV34	1650	rock debris	8.09.2009	1/0
Beskid Wyspowy (Western Beskid Mts)	Żmiącki stream	49°43′38″ N; 20°29′38″ E	DA60	670	Alder and sycamore forest	3.05.2007	2/1
						6.09-7.10.2007	1/0

Table S4. New localities of *Micrargus herbigradus* (ordered according to regions, eastwards). UTM – Universal Transverse Mercator, m – males, f – females

Geographic area	Locality	GPS Coordinates	UTM Square	Altitude (m a.s.l.)	Habitat	Dates (exposure time for pitfall traps or precise date)	Total number m/f
Lubusz Voivodeship	Owczary	52°'28'53" N; 14°37'50" E	VU71	16	xerothermic grasslands	4-29.04.2010	1/0
Izera Mts	Polana Izerska	50°52'33" N; 15°20'42" E	WS23	970	transition mire	10-24.05.2010	2/0
						24.05-14.06.2010	4/0
						14.06-2.07.2010	1/0
						22.04-10.05.2011	0/1
						10.05.2011	0/1
	Biała Droga	50°52'23" N; 15°21'23" E		965		10-24.05.2010	1/1
						24.05-14.06.2010	2/0
						14.06-02.07.2010	1/0
						02-23.07.2010	1/0
						23.07-11.08.2010	1/1
						10-29.05.2011	1/0
						29.05-15.06.2011	0/1
	Podmokła	50°51'53" N; 15°21'39" E		915	bog	23.07-11.08.2010	0/1
	Hala Izerska	50°51'07" N; 15°21'39" E		835		10.08-01.09.2010	0/1
						23.04-09.05.2011	1/0
						30.08-20.09.2011	1/0
						26.04-08.05.2012	1/0
	Kobyła Stream/Izera River	50°50'24" N; 15°22'10" E		835	dwarf mountain pine	28.05-13.06.2012	1/0
		50°50'25" N; 15°22'14" E		840	grasslands	26.04-08.05.2012	1/1
						08-28.05.2012	1/1
						13.06-03.07.2012	1/0
						22.07-10.08.2012	1/1
						10-29.08.2012	0/1
		50°50'11" N; 15°22'26" E		840	transition mire	25.04.2010	0/1
						09.05.2010	0/1
						13.06-01.07.2010	1/0
						01-22.07.2010	0/1
	slopes of Sine Skałki	50°51'29" N; 15°22'36" E		870		10-24.05.2010	1/0
		50°51'22" N; 15°23'27" E		970	spruce forest	09-29.05.2012	1/0
Giant Mts	Jakuszyce Pass, Bagnisko	50°49'31" N; 15°26'35" E	WS33	855	regenerating bog	28.05-12.06.2010	0/3
						12.06-30.06.2010	3/0
						30.06-21.07.2010	1/0
						16.09-16.10.2010	0/1
						24.04-08.05.2011	1/1
						08-27.05.2011	0/1
						27.05-13.06.2011	1/0
						13.06-02.07.2011	0/1
	Kamiennik/Mumlawski Pass	50°47'40" N; 15°28'40" E	WS32	1215	raised/transition mire	19.07-09.08.2010	0/1
						25.04-08.05.2011	0/1
						02-19.07.2011	0/1
						07-27.05.2012	1/0

Micrargus herbigradus cont.

Geographic area	Locality	GPS Coordinates	UTM Square	Altitude (m a.s.l.)	Habitat	Dates (exposure time for pit fall traps or precise date)	Total number m/f
Giant Mts	Szrenica/Kamiennik Pass	50°47'20" N; 15°30'01" E	WS32	1250	subalpine bog	12.06.2010	0/1
						28.05-12.06.2010	0/1
						13.06-02.07.2011	1/0
	Kocioł Szrenicki	50°47'17" N; 15°31'50" E		1170	sloping poor fen	09-28.08.2011	1/0
		50°47'01" N; 15°31'52" E		1300		19.07-09.08.2011	0/1
	by Wężówka	50°48'23" N; 15°35'39" E	WS42	775	transition mire	06-26.05.2012	0/1
		50°48'21" N; 15°35'39" E		780	spruce forest	17.04-06.05.2012	4/0
						06-26.05.2012	1/1
						14.06-05.07.2012	1/1
						05-25.07.2012	1/0
						25.07-12.08.2012	1/1
						22.09-20.10.2012	0/1
	Polana	50°46'01" N; 15°42'13"E		1075	transition mire	12.05.2011	0/2
						16.06-03.07.2010	1/0
						21.09-13.10.2010	0/1
	Równia pod Śnieżką	50°44'24" N; 15°42'24" E		1430	subalpine *Nardus stricta* grasslands	15.06.2012	0/1
						11-31.05.2012	1/0
						23.09-19.10.2012	0/1
Lower Silesian Wilderness	Przemków Landscape Park	51°28'04" N; 15°42'07" E	WT40	150	heather	08-29.05.2015	0/1
						01-21.08.2015	0/1
Pomeranian Lakeland	'Stary Załom' nature reserve	53°'05' N; 16°03' E	WU78	60	dry meadows	30.05-28.06.2012	0/1
						20.05-28.06.2013	1/0
The National Park of Góry Stołowe	Wielkie Torfowisko Batorowskie	50°27'27" N; 16°22'49" E	WR99	715	peat bog	19.05-03.06.2010	1/0
						03-19.06.2010	1/1
Owl Mountains (Central Sudetes)	Wielka Sowa	50°39′ N; 16°30′ E	XS01	ca 910	120 year old declining upper montane spruce forest	4.07-1.08.2005	1/0
Wielkopolsko-Kujawska Lowland	Góra by Czarnków	52°'52'51" N; 16°29'47" E	XU06	80	xerothermic grasslands	25.04-30.05.2012	1/0
						1-19.05.2013	0/1
	Biedrusko	52°33'17" N; 16°56'09" E	XU32	79	alder forest	31.05-3.07.2012	1/0
	'Ostnicowe Parowy Gruczna' nature reserve	53°20"34" N; 18°18'09" E	CE21	62	xerothermic grasslands	28.05-06.07.2011	1/0
	Unisław	53°'12'56" N; 18°22'39" E	CD29	48		26.04-28.05.2011	2/0
						28.05-06.07.2011	2/0
						22.03-24.04.2012	1/0
						24.04-30.05.2012	0/1
	Włocławek	52°'39'43" N; 19°07'58" E	CD73	47		30.05-27.06.2012	1/0
Silesian Voivodeship	Rybnik	50°07' N; 18°33' E	CA25	240	alder forest	-	0/1
						03.06.2016	1/0

Micrargus herbigradus cont.

Geographic area	Locality	GPS Coordinates	UTM Square	Altitude (m a.s.l.)	Habitat	Dates (exposure time for pitfall traps or precise date)	Total number m/f
Orawa-Nowy Targ Basin	'Pustać' by Chyżne	49°24′45″ N; 19°43′41″ E	DV07	692	peat bog, hollow	08–21.08.2008	1/0
		49°24′46″ N; 19°43′36″ E			peat bog	14–27.06.2008<br>20.10.2008-4.04.2009	0/1<br>1/0
	'Jasiowska Puścizna' by Jabłonka	49°27′24″ N; 19°45′52″ E	DV17	650	peat bog, hollow	04–14.07.2008	1/0
		49°27′27″ N; 19°46′11″ E			meadows		1/0
	'Baligówka' by Czarny Dunajec	49°28′02″ N; 19°48′38″ E	DV18	653	peat bog	02–18.05.2008<br>09.04-9.05.2009	1/1<br>1/0
		49°27′58″ N; 19°48′38″ E		652	peat bog, hollow	07–28.06.2008	0/1
	Zakopane, Harenda	49°19′ N; 19°58′ E	DV26	895	spruce forest	04.06.2011	2/2
	Cisowa Skała	49°26'11" N; 20°06'20" E	DV37	640	xerothermic shrubs and forest edge	18.04.2010	3/8
Gorce Mts	Polana Szlagowa	49°31'55" N; 20°11'09" E	DV48	1050	young spruce and meadow	03.09.1986	6/4
	Polana Magurki	49°31' N; 20°11' E		1000	mountainous meadow	03.09.1986	1/0
	Ochotnica Górna	49°30' N; 20°12' E		615	by buildings	05.09.1986	1/0
Świętokrzyskie Voivodeship	Radoszyce	51°04' N; 20°16' E	DB45	230	-	14.04.2015	1/0
Masurian Lakeland	Jaroty by Olsztyn	53°43'55" N; 20°27'15" E	DE65	122	moist forest	21.04.2008<br>05.05.2008<br>22.09.2008	1/0<br>6/0<br>0/1
	'Mszar' nature reserve by Olsztyn	53°48'54" N; 20°27'41" E	DE66	114	peat bog	23.07.2010	1/0
	at the Łuknajno Lake	53°48'24" N; 21°38'30" E	EE46	120	alder forest	10.04-03.05.2007	1/0
	by Urwitałt	53°48'13" N; 21°38'42" E		120	meadow	10.04-03.05.2007<br>03-24.05.2007<br>24.05-16.06.2007	1/0<br>1/0<br>0/1
		53°48'34" N; 21°39'37" E		135	peat bog (1)	03-24.05.2007	1/0
		53°48'57" N; 21°40'19" E		130	peat bog (2)		1/0
Beskid Wyspowy (Western Beskid Mts)	Żmiącki Stream	49°43′38″ N; 20°29′38″ E	DA 60	670	Alder and sycamore forest	03.05.2007	2/1
	Klęczany	49°40'36" N; 20°36'58" E	DA70	410	former stone quarry	02.05.2007	1/0
Mazovian Lowland	'Ponty Dęby' nature reserve	51°30′54″ N; 21°23′03″ E	EC20	165	old oak forest	02–29.04.2017	1/2
Sandomierz Basin	Malinie	50°21'06" N; 21°27'33" E	EA37	167	city park	08–22.05.2016	1/0
	Kotowa Wola	50°36'03" N; 21°56'05" E	EB60	152	young pine forest	14.05-4.06.1998<br>06–21.07. 1998	2/0<br>1/0
	'Czarny Las' forest district	50°28" N; 22°08" E	EA 89	170	oak forest	01–14.05.1998	1/0
	Wola Zarczycka	50°18" N; 22°15" E	EA87	ca 200	in nest of *Lasius fuliginosus*, pine forest	14.08.2005<br>21.08.2005<br>23.10.2005	0/1<br>0/2<br>0/1
	Bolestraszyce, remnants of the 'San Riedau' fort	49°49'19" N; 22°51'38" E	FA32	213	black elder and black locust shrubs	22.05.2014	6/4
Małopolska Upland	'Góry Pieprzowe' nature reserve	50°41" N; 21°47'E	EB51	150	xerothermic shrubs	20.06-3.07.2008<br>17.05-25.06.2009<br>1.06.2008<br>12.10.2008	3/1<br>4/1<br>0/2<br>0/1

Micrargus herbigradus cont.

Geographic area	Locality	GPS Coordinates	UTM Square	Altitude (m a.s.l.)	Habitat	Dates (exposure time for pit fall traps or precise date)	Total number m/f
Lublin Upland	'Skarpa Dobrska' nature reserve	51°16'50" N; 21°53'44" E	FB68	165	xerothermic shrubs	04.04.2009<br>24.04.2009	0/1<br>2/1
	Parchatka	51°21'55" N; 22°00'10" E	FB69	165	loess ravine	26.04.2004	3/1
	Krężnica Jara	51°08' N; 22°28' E	FB06	190	mixed forest	10.11.2004	1/2
	Lublin, Botanical Garden	51°15' N; 22°30' E	EB08	206	shrubs	03.05.2015	2/1
	Lublin, Czuby	51°13' N; 22°32' E	EB07	180	storm drain canal	15.12.2004	0/1
	Suchodoły by Fajsławice	51°04' N; 22°58' E	FB36	215	plantation of lemon balm (*Melissa officinalis*),	25.05–3.06.2005	0/1
					plantation of common marigold (*Calendula officinalis*),	14–29.06.2005<br>12–25.08.2005	1/0<br>1/0
	Kępa	51°10' N; 23°36' E	FB87	174	agrocenosis	? –28.V.2013	0/1
	Gródek by Hrubieszów	50°47'10" N; 23°56'43" E	GB03	185	xerothermic grasslands	06.06.2001<br>05.10.2001<br>16.07.2002	1/0<br>1/0<br>1/0
	Kryłów	50°40'48" N; 24°03'40" E	KS91	184	old park	04.04.2002	1/0
Eastern Beskids	Węgierka by Rożwienica	49°51′49″ N; 22°35′08″E	FA12	273	thistle meadow	06–12.05.2007<br>08–28.06.2007	1/1<br>1/0
	Kopysno	49°41'03" N; 22°38'49" E	FA10	455	blackthorn shrubs	05.06.2015	2/3
	'Salis Soglio' fort	49°45'26" N; 22°53'46" E	FA31	292	shrubs	24.05.2014	3/2
Roztocze	vincity of Bełżec	50°23′03″ N; 23°24′17″ E	FA78	278	dry pine forest (ca 40 years old)	01.05-16.05.2005	1/0
Polesie	Włodawa	51°33′ N; 23°33′ E	FC71	ca 170	in grass	? –16.07.2014	0/1
